# Systematic review on post-stroke computerized cognitive training: Unveiling the impact of confounding factors

**DOI:** 10.3389/fpsyg.2022.985438

**Published:** 2022-12-12

**Authors:** Paloma E. Fava-Felix, Silvia R. C. Bonome-Vanzelli, Fabiana S. Ribeiro, Flávia H. Santos

**Affiliations:** ^1^Department of Psychology, São Paulo State University, UNESP, São Paulo, Brazil; ^2^Department of Social Sciences, University of Luxembourg, Esch-sur-Alzette, Luxembourg; ^3^School of Psychology, University College Dublin, Dublin, Ireland

**Keywords:** aging, cerebrovascular disease, stroke, cognitive reserve, cognitive rehabilitation, neuropsychological rehabilitation

## Abstract

**Background:**

Stroke is a highly incapacitating disease that can lead to disabilities due to cognitive impairment, physical, emotional, and social sequelae, and a decrease in the quality of life of those affected. Moreover, it has been suggested that cognitive reserve (patients’ higher levels of education or having a skilled occupation), for instance, can promote faster cognitive recovery after a stroke. For this reason, this review aims to identify the cognitive, functional, and behavioral effects of computerized rehabilitation in patients aged 50 years or older who had a stroke, considering cognitive reserve proxies.

**Methods:**

We followed the Preferred Reporting Items for Systematic Reviews and Meta-Analysis—PRISMA, and performed the search for peer-reviewed randomized controlled trials without a date restriction on CINAHL, LILACS, PubMed, Scopus, and Web of Science databases were chosen.

**Results:**

We screened 780 papers and found 19 intervention studies, but only 4 met the inclusion criteria and shared data. These studies included computerized tools for motor and cognitive rehabilitation in the experimental groups. In all studies, computerized training was combined with other interventions, such as standard therapy, occupational therapy, and aerobic exercises. There were 104 participants affected by ischemic or hemorrhagic stroke, predominantly male (57.69%), and all with cognitive impairment.

**Conclusion:**

Despite a limited number of studies, varied methods and insufficient information available, schooling as a CR proxy combined with high-intensity computerized cognitive training was key to mediating cognitive improvement. The systematic review also identified that the associated ischemic stroke and shorter time of onset for rehabilitation contribute to the cognitive evolution of patients. Findings do not support a greater benefit of computerized cognitive training compared to conventional cognitive therapies.

**Systematic review registration:**

[https://www.crd.york.ac.uk/prospero/display_record.php?RecordID=296193], identifier [CRD42022296193].

## Introduction

According to the World Health Organization, as of 2015, life expectancy increased worldwide to a mean of 63.1 years old. In comparison to 50 years ago, people are living on average 20 years more, which may determine a longer retirement (World Health Organization, 2015). The mean age for productive (working) life is 62 years old in 12 countries studied (9 countries in Europe, the United States, Canada, and Japan) by Börsch-Supan and Coile (2018), which expresses the importance of effective rehabilitation of patients who suffered a stroke, allowing them to productively work and have a better quality of life.

The increase in life expectancy creates healthcare challenges due to aging-related diseases, such as stroke (Wafa et al., 2020; Nie et al., 2022). Stroke is a focal neurological deficit with rapid advancement arising from an injury caused by an interruption of the blood supply in the injured brain region (Albers et al., 2002). Strokes are divided into two categories, ischemic and hemorrhagic. The ischemic is the most frequent and presents the obstruction of a vessel. The mechanism of the injury is subdivided into atherosclerosis of large arteries and cardioembolic or small vessel diseases. The hemorrhagic one presents a blood vessel rupture and is more severe and fatal (Petty et al., 1999; Grysiewicz et al., 2008). In addition, there is the Transient Ischemic Attack, an event that occurs for less than 24 h with the momentary interruption of a vessel. The Transient Ischemic Attack shows focal symptoms, lasts about an hour, and, without effective infarction (Albers et al., 2002), is considered a potential predictor of stroke (Johnston et al., 2000).

Stroke is the second leading cause of death worldwide, being the leading cause of disability in adults and is commonly connected to dependence and dementia (Feigin et al., 2014; McHutchison et al., 2017). The global burden and prevalence of stroke are progressive. Among the 16.7 million people annually affected by it in the world, 1/3 will not survive, and another 1/3 will present some cognitive impairment or dementia (Hachinski and World Stroke Organization, 2015; McHutchison et al., 2017). Among patients with stroke, older adults are those with an increased risk of disability and mortality, in which the ischemic type is more frequent than the hemorrhagic one (Winovich et al., 2017).

Some investigations show that Cognitive Reserve (CR) proxies can provide functional strategies and an increased efficiency in cognitive processing, enabling greater resistance to brain damage (Stern, 2002). In this sense, CR proxies can interact and positively mediate the effects of brain damage in strokes (Umarova et al., 2019). For instance, regarding schooling, Ojala-Oksala et al. (2012) conducted a study with 486 patients who had an ischemic stroke. They pointed out that this proxy was an effective mediator of the outcome after an ischemic stroke. The study by Umarova et al. (2019) investigated the influence of CR, through schooling, in the cognitive recovery of patients with ischemic stroke in the right hemisphere (mean 13.4 ± 3.1). Schooling predicted the severity of cognitive deficits in working memory and executive functions in acute ischemia, regardless of age and lesion size.

CR is conceptualized as the relationship between the severity of brain damage and clinical manifestations, and it can be accessed in the face of functional impairment (Stern, 2021). More importantly, it can influence rehabilitation due to stroke-related damage (Ojala-Oksala et al., 2012; Umarova et al., 2019; Rosenich et al., 2020). CR derives from factors capable of shaping intellectual efficiency throughout life and depends on lifestyle factors that lead to individual differences in brain networks. Thus, it is understood that CR proxies (such as education, occupational level, and physical activities, etc.) contribute to the formation of more resistant neural networks that protect cognitive function, even in neuropathological progress (Stern, 2012). On the other hand, the Brain Reserve (Katzman, 1993) corresponds to the quantitative neural characteristics such as brain size or neuron count (Barulli and Stern, 2013) that the individual has to respond to brain damage caused, for example, by stroke.

Corroborating this, the meta-analysis of McHutchison et al. (2017) comprising 90 articles, including approximately 164,683 participants with stroke and more than 5 million without stroke, sought to explore the risk of stroke in adulthood associated with low education, low socioeconomic status and/or low intelligence quotient in childhood. Many studies have used years of schooling as a proxy for the intelligence quotient because the likelihood of pursuing higher education is greater among those with average or high intelligence quotient. In line, a higher socioeconomic status allows better access to education, health and safety, minimizing stroke risks. In addition, these factors are protective and moderately associated with greater brain resilience, preventing vascular damage (Stern, 2012, 2021). By contrast, low education, low socioeconomic level and/or low intelligence quotient increase the lifetime risk of stroke and, consequently, leads to a higher dementia risk (Galobardes et al., 2006; Addo et al., 2012; Stern, 2012; McHutchison et al., 2017).

In another review study with 27 articles, including cross-sectional, longitudinal, retrospective, and observational studies focused on identifying whether risk factors (demographic, clinical, psychological, and physical) could influence cognitive function among stroke survivors, the results showed that in more than half of the articles, the demographics variables (age, low levels of education, and history of stroke) were risk factors for cognitive impairment. In addition to comorbidities, such as diabetes mellitus and hypertension, also stroke characteristics, i.e., size and location of brain lesions, which interfered with cognitive functioning; as well as depression symptoms, were determining factors for cognitive impairment (Mohd Zulkifly et al., 2016).

Emotional factors, such as post-stroke depression, can also be manifested by fatigue, lack of interest, and physical pain, interfering with the rehabilitation process (Chen et al., 2022). Emotional, cognitive, and motor disorders remain a challenge for the rehabilitation process due to the heterogeneity of cases and their severity variations.

Their impact varies according to the level of premorbid functioning (Umarova et al., 2019; Chen et al., 2022). Besides, lesions are not identical, and even brain injury in the same location can affect different abilities and be associated with different levels of recovery (Stern et al., 1994; Stern, 2009, 2021).

Among survivors, stroke causes motor sequelae (e.g., aphasia, apraxia, negligence) (Larivière et al., 2018; McHutchison et al., 2019) and cognitive impairment (e.g., attention, memory and executive function deficits) (McHutchison et al., 2019; Demeyere et al., 2021), both interfering with the subjects’ quality of life, which can lead to comorbidities such as depression (McHutchison et al., 2019).

Due to the frequent physical and cognitive impairment, training in both aspects is essential to improve the independence of those affected (Zhang et al., 2021). Many survivors acquire physical sequelae, interfering with the performance of daily activities (Ozen et al., 2021). Adapting intervention strategies to each patient is essential to improve balance, endurance, and mobility (Prout et al., 2015). Physiotherapy and cognitive remediation, for example, combined or isolated, are mostly aimed at relearning motor skills and improving quality of life (Kleim and Jones, 2008; Levin et al., 2015).

Regarding the best clinical practices in post-stroke rehabilitation of adults, there is evidence that early multidisciplinary rehabilitation is beneficial, as they often involve occupational therapy, physical therapy, and speech and language therapy, which can occur as soon as the patient is able to withstand them (Winstein et al., 2016; Tsao et al., 2022). Furthermore, research indicates that early intervention is essential (Bernhardt et al., 2008; Yagi et al., 2017), especially for the ischemic type (Liu et al., 2021). However, there is no consensus on the ideal time to start the rehabilitation. For some researchers, it can take place within 24–72 h of the onset of the stroke (Bernhardt et al., 2008; Aries et al., 2013; AVERT Trial Collaboration Group, 2015). In turn, the study by Liu et al. (2021) differentiates between early rehabilitation starting between 72 h to 7 days after the onset of a stroke and ultra-early rehabilitation: the one which starts within 72 h.

Post-stroke neuropsychological rehabilitation refers to several strategies tailored to each patient’s clinical and personal features, aiming to improve cognitive, emotional, and psychosocial functioning. It includes paper and pencil cognitive training and home-based activities, among other resources applied individually or in small groups. Neuroimaging studies indicate that neuropsychological interventions produce neuroplasticity, for instance, executive functions training stimulates pre-established brain foci (Burgess and Alderman, 2013).

While effective, conventional cognitive training lacks precise progression control (strict number of attempts, types of errors, time spent on tasks, etc.), it does not provide instant feedback (Levin et al., 2015) and may not be engaging and challenging as training embedded in computational tools (Zhou et al., 2022). Computerized cognitive training has shown promise in post-stroke, is often used in individual sessions of specific programming with adaptive, repetitive, and standardized exercises and provides storage of patients’ progress and instant feedback, in addition to being motivating (Cumming et al., 2013; Verstraeten et al., 2016; Mingming et al., 2022; Zhou et al., 2022).

The systematic review and meta-analysis by Nie et al. (2022), with 32 randomized clinical trial studies including 1,837 participants, investigated the effectiveness of computerized cognitive rehabilitation compared to conventional treatment and nursing and/or health education for the primary disease in patients with post-stroke cognitive impairment. The patients showed significant improvement in global cognition and activities of daily living. The Loewenstein Cognitive Assessment of occupational therapy was the screening measure used in six studies comprising 318 patients and identified significant improvement of the latter after computerized cognitive training. Furthermore, regarding activities of daily living, four studies evaluated by the Barthel Index and four studies by the Modified Barthel Index, showed better outcome measures compared to the control group. Therefore, by targeting motivation and adherence to rehabilitation, computer-assisted cognitive interventions are proving effective for post-stroke patients (Nie et al., 2022).

The meta-analysis by Sun et al. (2021) showed significant benefits of combined cognitive exercise interventions (more specifically, cognitive behavioral training, computer-assisted cognitive training and physical training) compared to no intervention, delayed intervention, sham intervention, or passive training in patients with cognitive impairment post-stroke in executive, attention, and memory functions. In addition, another review that investigated the effects of aerobic exercise combined with cognitive training (mostly computerized) found that the combined intervention was associated with post-intervention improvement on at least one cognitive test across all included studies (Amorós-Aguilar et al., 2021).

This systematic review seeks to identify cognitive, functional, and behavioral effects of cognitive training in patients with stroke aged 50 years or older, considering cognitive reserve proxies. Having the following question as the basis: *what are cognitive training’s cognitive, functional and behavioral effects in stroke patients over 50 years of age, considering cognitive reserve proxies?* Specifically, this research aims to scrutinize how and in which contexts CR proxies such as educational level, socioeconomic level and/or regular physical activity can influence the cognitive results in the rehabilitation measures. Considering clinical evidence, we hypothesize that proxies such as schooling, occupational level, and physical activity are mediating factors of the rehabilitation process. We also aim to verify whether clinical variables, such as stroke severity and age of occurrence, impact these results, taking into account the results of the interaction between group and intervention time (duration, intensity, and frequency) of randomized control trial studies focused on post-traumatic rehabilitation after a stroke.

Taking into account that no reviews have explored the influence of diverse CR proxies on training outcomes cognitive (Cumming et al., 2012; Pang et al., 2013; McHutchison et al., 2017; Penna et al., 2021; Chen et al., 2022; Mugisha et al., 2022; Nie et al., 2022; O’Donoghue et al., 2022; Wiley et al., 2022), this research is essential to identify which patients benefit the most from the intervention and to guide the development of intervention programs, investment in equipment, research funding, and to identify the quality of life markers for patients with stroke.

## Materials and methods

The systematic review was registered in PROSPERO—International Prospective Register of Systematic Reviews (CRD42022296193) and follows the PRISMA guidelines—Preferred Reporting Items for Systematic Reviews and Meta-Analysis (Page et al., 2021), to ensure rigor and replicability (Moher et al., 2009). It is organized into the following steps: research question, search strategies and literature search, studies selection, data extraction, evaluation of the methodological quality of the studies, data synthesis, evaluation of the quality of evidence, and writing and publication of results.

The risk of bias for each study was evaluated using the Revised Cochrane Risk of Bias tool for Randomized Trials—RoB 2.0 (Higgins et al., 2022).

### Search strategy and selection of studies

The databases CINAHL, Lilacs, PubMed, Scopus, and Web of Science were selected to search for peer-reviewed articles without date restrictions. The search terms used were: (stroke OR cerebrovascular disease OR vascular disease OR vascular brain disorders) AND (brain OR brain injury) AND (cognitive reserve OR cognit* OR cognitive function) AND (rehabilit* OR cognitive rehabilitation OR cognitive training OR rehabilitative treatment OR rehabilitation neuropsyc*) NOT (pharmacol*) AND (cognitive assessment OR cognitive scales) AND (adult OR older adult), adapted according to each database.

All article titles and abstracts were independently selected by two authors (PF-F and SB-V). Then, the articles were imported into Excel^®^ (version 2019), and the duplicated ones were deleted. Afterward, the selected articles were read in full to verify the eligibility criteria. The existence of discrepancy was resolved with the help of the third author (FR). After selection, the data was organized in an Excel^®^ spreadsheet.

### Eligibility criteria

Inclusion criteria: (i) randomized control trials that include neuropsychological evaluation pre- and post-intervention; (ii) hemorrhagic, ischemic, and transient stroke as well as frequency of occurrence; (iii) inclusion of any type of cognitive training, i.e., computerized or conventional; (iii) patients over 50 years of age; (iv) cognitive reserve proxies (one or more CR proxies, such as physical activity, education and/or occupation level). Only articles in English were included due to the fact that more than 90% of the articles indexed in the field of the natural sciences are published in English (Hamel, 2007; Ammon, 2012), increasing the probability of citations and articles impact in the scientific community (Di Bitetti and Ferreras, 2017).

The exclusion criteria are: (i) randomized studies with children and adolescents; (ii) theoretical projects such as systematic reviews, and (iii) duplicated studies and theses. The outcome measure was the interaction between group and time presented in cognitive tests and behavioral scales.

### Data collection and analysis

It was sought to classify patients for analysis according to their type of stroke (hemorrhagic, ischemic, or transient).

According to the available data on the different computerized cognitive training methods, the following topics were examined: the time of involvement and the beginning of training; duration and number of sessions; individual and group training responses. In addition, changes in pre-and post-intervention measures, comparison of results from control and experimental groups, or reported effect sizes were also analyzed.

In the randomized control trials, the existence of control groups with healthy patients was verified for possible surveys looking for differences (or not) between the affected and healthy groups. However, all the individuals included in the control groups had also suffered a stroke. Thus, the sociodemographic and clinical characteristics and outcome measures were verified.

Information from included studies was evaluated to determine whether CR proxies and the duration of intervention programs influence task performance.

These observations were carried out taking into account the data received via email and consulted in the articles. The types of intervention were confirmed (conventional versus computerized) and it was considered if they were either cognitive or associated with other activities (ex: physical activities, physiotherapy). We also analyzed them with sociodemographic data (sex, age) and clinical characteristics (type of stroke, hemisphere, and onset time).

Due to the small number of articles, the heterogeneity of the data, the differences between participants, and the configuration of the studies, it was not possible to perform the meta-analysis. However, it is possible to gather studies for a systematic review without performing a meta-analysis, which is considered a legitimate choice (Ioannidis et al., 2008).

## Results

### Study selection process

**FIGURE 1:**
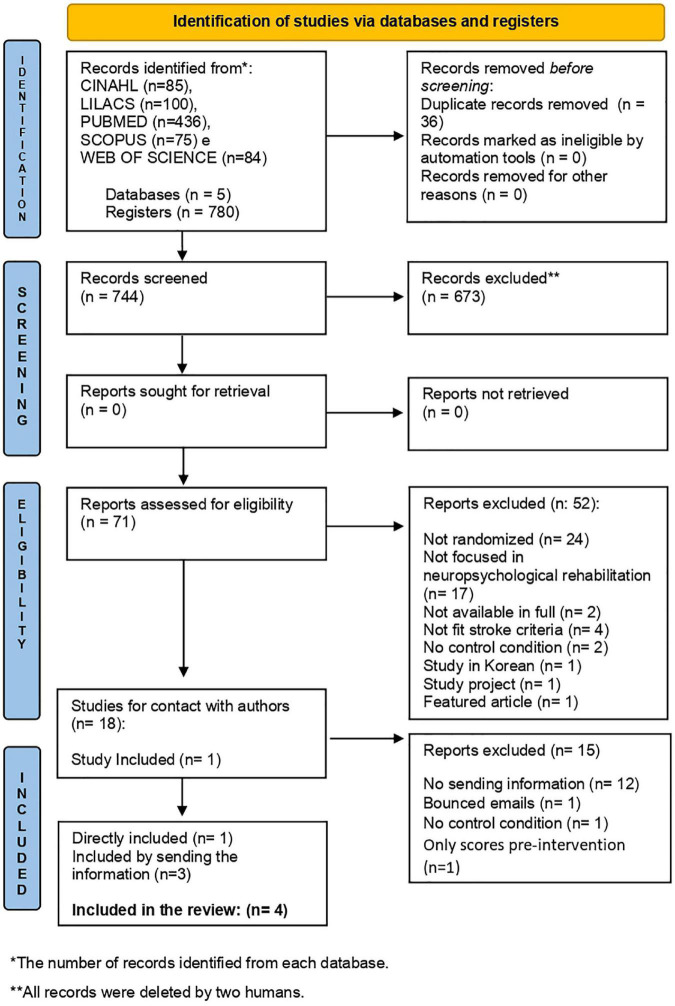
Flow diagram: research and selection process according to PRISMA guidelines.

The selection of studies started on January 6th and ended on January 12th, 2022, with 780 articles accessed through the five chosen databases. Thirty-six articles were excluded due to duplication, and 744 studies were included for titles and abstract reading. A total of 673 fthe articles, 19 were identified with potential for the sample (Figure 1), but only one article (Prokopenko et al., 2013) could be selected directly, as their investigation fully met the criteria.

Then, we contacted the authors representing the other 18 articles by email, reporting the review proposal and requesting access to information concerning 50-year-old patients and above (gender, CR proxies, and pre-and post-neuropsychological assessment results). Five authors out of the 18 articles replied to the email, but only 3 articles were included, with 15 studies excluded in this process (exclusions detailed in Table 1). The entire stage of contacting and receiving information lasted about 21 days, but no response was obtained.

**TABLE 1:** Characteristics of studies that were contacted and then excluded.

References	Sample	Participants’ age criteria	Cognitive reserve	Required data absent from published paper	Contacting authors by email
Aben et al. (2013)	*N* = 153	Between 18 and 80 years	Education and occupation	Demographic data, clinical features of stroke, and pre-and post-intervention assessment results of only individuals aged 50 years and older	All forwarded emails have been bounced
Chen et al. (2015)	*N* = 80	Between 45 and 74 years	Education and occupation	Demographic data, clinical features of stroke, and pre-and post-intervention assessment results of only individuals aged 50 years and older	No return
De Luca et al. (2018a)	*N* = 35	Mean age of included: 43.15 ± 16.85 years	Education	Demographic data, clinical features of stroke, and pre-and post-intervention assessment results of only individuals aged 50 years and older	No return
De Luca et al. (2018b)	*N* = 12	Mean age of included: 40 ± 14 years	Education	Demographic data, clinical features of stroke, and pre-and post-intervention assessment results of only individuals aged 50 years and older	No return
Jiang et al. (2016)	*N* = 204	Between 18 and 75 years	Education	Demographic data, clinical features of stroke, and pre-and post-intervention assessment results of only individuals aged 50 years and older	No return
Oh et al. (2019)	*N* = 31	Between 20 and 85 years	Education	Demographic data, clinical features of stroke, and pre-and post-intervention assessment results of only individuals aged 50 years and older	No return
Ozen et al. (2021)	*N* = 30	Between 18 and 85 years	Education	Demographic data, clinical features of stroke, and pre-and post-intervention assessment results of only individuals aged 50 years and older	No return
Pereira et al. (2021)	*N* = 20	Mean age included: 60.4 ± 8.2 years (range 31–71 years)	Education (reading and writing)	Demographic data, clinical features of stroke, and pre-and post-intervention assessment results of only individuals aged 50 years and older	Return (cross-sectional studies)
Ranzani et al. (2020)	*N* = 27	Between 18 and 90 years (range 38–85 years)	Requested by email	Demographic data, clinical features of stroke, and pre-and post-intervention assessment results of only individuals aged 50 years and older	No return
Richard et al. (2020)	*N* = 54	Mean age of included: 69.72 ± 7.46 (range 47.8–82.0 years)	Education	Demographic data, clinical features of stroke, and pre-and post-intervention assessment results of only individuals aged 50 years and older	Return (only have MoCA scores pre-intervention)
Rogers et al. (2019)	*N* = 21	Over 18 years	Education	Demographic data, clinical features of stroke, and pre-and post-intervention assessment results of only individuals aged 50 years and older	No return
Torrisi et al. (2019)	*N* = 40	Mean age of included: 55.2 ± 18.4	Education	Demographic data, clinical features of stroke, and pre-and post-intervention assessment results of only individuals aged 50 years and older	No return
van de Ven et al. (2017a)	*N* = 97	Between 30 and 80 years	Education	Demographic data, clinical features of stroke, and pre-and post-intervention assessment results of only individuals aged 50 years and older	No return
van de Ven et al. (2017b)	*N* = 97	Between 30 and 80 years	Education	Demographic data, clinical features of stroke, and pre-and post-intervention assessment results of only individuals aged 50 years and older	No return
Wilson et al. (2021)	*N* = 17	Over 18 years	Requested by email	Demographic data, clinical features of stroke, and pre-and post-intervention assessment results of only individuals aged 50 years and older	No return

Table 1 presents the 15 studies that did not meet the age criteria, including patients under 50 years of age in their samples. However, in 12 studies (Chen et al., 2015; Jiang et al., 2016; van de Ven et al., 2017a,b; De Luca et al., 2018a,b; Oh et al., 2019; Rogers et al., 2019; Torrisi et al., 2019; Ranzani et al., 2020; Ozen et al., 2021; Wilson et al., 2021) we did not get a response; in 1 study (Aben et al., 2013) forwarded emails were bounced, and in 2 studies (Richard et al., 2020; Pereira et al., 2021) we received feedback via email, but access to information only was possible for the experimental group.

We selected 3 articles with information received via email and one article that met all the criteria from the beginning of the selection. The final sample of the systematic review consisted of 4 articles. The following are presented: the authors, year of publication, country of study, number of participants selected for this systematic review and electronic address.

– Faria et al. (2018), Portugal (n:22). Frontiers in Psychology;
– Kotov et al. (2020), Russia (n:23). Neuroscience and Behavioral Physiology;
– Prokopenko et al. (2013), Russia (n:43). Journal of the Neurological Sciences;
– Yeh et al. (2019), Taiwan (n:16). Archives of Physical Medicine and Rehabilitation;

The article’s search and selection process details are shown in the flowchart in Figure 1.

### Quality assessment of included studies

Regarding the methodological assessment, none of the four studies included had a high risk of bias. In the dimensions of randomization bias (D1), bias due to deviations from intended interventions (D2), bias due to lack of data in the results (D3), and bias due to outcome measurements (D4), all studies had a low risk of bias. In the assessment of bias due to the selection of the reported outcome (D5), two studies (Faria et al., 2018; Yeh et al., 2019) maintained a low risk of bias and two (Prokopenko et al., 2013; Kotov et al., 2020) were evaluated with some concerns.

**FIGURE 2:**
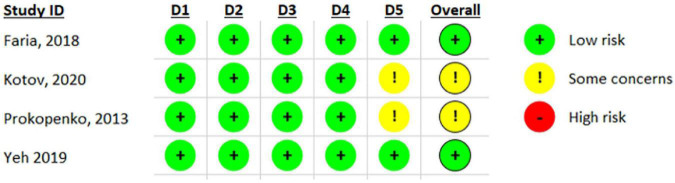
Risk of bias analysis with individual results for each domain of included studies through RoB 2.0.

**FIGURE 3:**
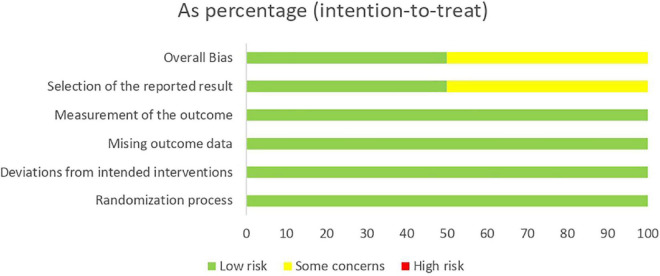
Risk of bias of randomized clinical trials included and evaluated through RoB 2.0.

The risk of bias assessment is presented in Figures 2, 3.

### Research objective

Since the objectives of our review were to identify the effects of cognitive training in stroke patients aged 50 years or older, considering cognitive reserve proxies, also verifying whether clinical variables impacted these results, the studies included combined computerized cognitive training with other rehabilitation modalities, namely standard therapy (Prokopenko et al., 2013; Kotov et al., 2020), conventional occupational therapy (Faria et al., 2018) and training aerobic exercise (Yeh et al., 2019).

The CR proxies reported by the authors were occupational level (Faria et al., 2018) and predominant education (Prokopenko et al., 2013; Faria et al., 2018; Yeh et al., 2019). The years of schooling acted as a mediator of change. Regarding the duration and intensity, we observed variation among studies, as shown in Table 2, but also dependent on the type of the intervention, for instance, Reh@Task, 1 month, 3 times a week (Faria et al., 2018) lasted more than BrainHQ, 12 to 18 months, 2 or 3 times a week (Yeh et al., 2019).

**TABLE 2:** Intervention characteristics.

References	Computer system	Intervention	Duration of training	Number of sessions/Intensity (*I*)
Faria et al. (2018)	Reh@Task	Experimental. Conventional occupational therapy and multifunctional virtual reality.	1 month	12 sessions *I* = 3 times/week
	Without Reh@Task	Control. Standard occupational therapy, spatial and temporal orientation activities, and writing training.	1 month	12 sessions *I* = 3 times/week
Kotov et al. (2020)	Complex multimodal stimulation program that included BCNI	Experimental. Standard therapy (medication, speech therapy, physical therapy and therapeutic exercises), complex multimodal stimulation with BCNI, cognitive training, stabilometric exercises and vibration therapy.	2 months to 2 years	BCNI: 3 sessions TV: 8–10 sessions *I* = Not specified
	BCNI	Control. Standard therapy (medication, speech therapy, physical therapy and therapeutic exercises) and complex multimodal stimulation with BCNI.	2 months to 2 years	BCNI: 3 sessions I = Not specified
Prokopenko et al. (2013)	Computer programs	Experimental. Standard treatment in the inmate rehabilitation department and computer programs.	2 weeks	PC:15 sessions I = 7 times/week
	No computer programs	Control. Standard treatment in the inmate rehabilitation department	2 weeks	Not specified
Yeh et al. (2019)	Platform BrainHQ	Experimental. Aerobic exercise training (progressive resistance stationary bike) and computer-based cognitive training.	12–18 weeks	36 sessions *I* = 2 or 3 times/week
	Without BrainHQ	Control. Non-aerobic exercise training (flexibility exercises, muscle strengthening and balance training) and unstructured mental activities that did not train a specific cognitive domain (reading newspapers or magazines or watching videos related to health).	12–18 weeks	36 sessions *I* = 2 or 3 times/week

BCNI, brain-computer neural interface with an arm exoskeleton; BrainHQ, an online brain gym scientifically designed to train and improve the brain and its cognitive functions; TV, vibration therapy is a special rehabilitation device; PC, computer programs developed by the study group itself.

Considering the combination of schooling, intense computerized training and short time intervals for intervention after the stroke, better post-intervention change scores (MoCA) were identified in the experimental groups (Prokopenko et al., 2013; Faria et al., 2018).). However, it was not possible to identify disparities between rehabilitation involving computerized training (experimental group) and rehabilitation without computerized training (control group).

Finally, the reduced number of studies included in this review and the heterogeneity of the data did not meet the criteria for a meta-analysis (Noordzij et al., 2009).

### Characteristics of the selected studies

Table 3 summarizes the characteristics of the four articles selected for this systematic review. The study’s sample size varies from 16 to 43 participants, corresponding to 104 patients in total. All participants were diagnosed with stroke and presented with cognitive deficits. Regarding sex, the participants were predominantly male (57.69%). Among the groups, 55.73% were male in the experimental groups, and 60.46% were male in the control groups.

**TABLE 3:** General characteristics and outcome measures of the included studies.

References	Groups (*N*/mean age)	Gender (man/woman)	Education (years)	Type of stroke	Time months after stroke and beginning of rehabilitation	MoCA before mean results (DP)	MoCA after mean results (DP)	MoCA change points (DP)
Faria et al. (2018)	Experimental 11 (63)	7/4	6	11 Ischemic	1 Before 6 months 10 After 6 months	22 (4.75)	25.45 (2.50)	3 (3.17)
	Control 11 (69)	7/4	6	9 Ischemic 1 Hemorrhagic 1 Unknown	1 Before 6 months 8 After 6 months 2 Unidentified	20 (2.87)	23 (2.76)	3 (2.96)
Kotov et al., 2020)	Experimental 18 (60.83)	8/10	Not specified	16 Ischemic 2 Hemorrhagic	3 Before 6 months 15 After 6 months	24.83 (1.72)	26.44 (1.88)	1.61 (1.19)
	Control 5 (66)	3/2	Not specified	5 Ischemic	2 Before 6 months 3 After 6 months	23 (3.00)	25 (2.54)	2 (1.58)
Prokopenko et al. (2013)	Experimental 24 (63)	13/11	10	Not specified	2 weeks	20	25	5
	Control 19 (65)	9/10	10.1	Not specified	2 weeks	22	24	2
Yeh et al. (2019)	Experimental 8 (59.57)	6/2	9	Ischemic or Hemorrhagic	8 After 6 months	19.875 (5.33)	22.375 (6.43)	2.5 (2.44)
	Control 8 (64.40)	7/1	9,62	Ischemic or Hemorrhagic	8 After 6 months	19.375 (3.99)	19.875 (6.56)	0.25 (3.88)

Regarding age, the experimental groups had a lower mean than the control groups. Another detail is that three experimental groups (Prokopenko et al., 2013; Faria et al., 2018; Yeh et al., 2019) have higher pre- and post-intervention MoCA scores.

Education ranged from 6 to 10 years of study. However, one study (Kotov et al., 2020) did not describe the participants’ education in detail, as specified in Table 3.

The studies included: ischemic and hemorrhagic strokes. In the study by Faria et al. (2018) with 22 patients, 90.90% suffered an ischemic stroke, 4.54% a hemorrhage stroke and 4.54% were not identified. In the study by Kotov et al. (2020) with 23 patients, 91.30% had an ischemic stroke and 8.69% had a hemorrhagic stroke. The stroke type ratings were included in the studies by Prokopenko et al. (2013) and Yeh et al. (2019).

Regarding the injured brain hemispheres, two studies included the information. Specifically, in the study by Faria et al. (2018), of the 22 patients, 50% were injured on the left side of the brain, 40.90% on the right and 9.09% were not identified. In the Kotov et al. (2020) study, of the 23 patients, 60.86% were injured on the right side of the brain and 39.13% on the left side.

Regarding the CR proxies presented in Table 3, three studies (Prokopenko et al., 2013; Faria et al., 2018; Yeh et al., 2019) presented the level of education. Only one study (Faria et al., 2018) sent information about the participants’ occupational status. Studies are described here. In the experimental group there was housewife (*n* = 3); bricklayer (*n* = 1), plumber (*n* = 1), waiter (*n* = 1), butcher (*n* = 1), worker (*n* = 1), merchant (*n* = 1), unemployed (*n* = 1) and without identification (*n* = 1). The control group was composed by housewives (*n* = 3), fishermen (*n* = 2), driver (*n* = 2), grinder (*n* = 1), builder (*n* = 1), air force officer (*n* = 1) and unemployed (*n* = 1).

Regarding the occupational level proxy in the study by Faria et al. (2018), the four patients who presented the best screening measures (between 06 and 08 change points in the MoCA), after the intervention in the experimental group, were affected by ischemic stroke: plumber (male, 57 years old with 4 years of study), housewife (female, 55 years old with 6 years of schooling), retired (male, 78 years old with 4 years of schooling) and industrial worker (male, 64 years old with 7 years of schooling). The housewife was the one with the best measure of best.

In the control group, the three patients with the best screening measures (with 06 and 08 points of change in the MoCA) were affected by ischemic stroke: housewife (female, 76 years old with 4 years of schooling), grinder (male, 80 years old with 4 years of schooling) and constructor (man, 54 years old with 4 years of schooling. The housewife had the best measure of best.

The patient (man, 70 years old) with the highest level of education (17 years of study) in the experimental group, an officer in the Air Force, was affected by a hemorrhagic stroke and did not present any cognitive evolution.

The physical activity practice proxy was not presented in any study, referring to healthy habits. However, aerobic activity appears associated with computerized cognitive training as an intervention in the experimental group by Yeh et al. (2019), with the group showing improvement in the screening measure (MoCA). The three patients with the best cognitive alterations: were one man (68 years old with 8 years of schooling) and two women (58 years old with 12 years of schooling and 59 years old with 6 years of schooling). Occupational information was not provided.

Two studies (Prokopenko et al., 2013; Faria et al., 2018) with respectively 6 and 10 years of studies and interventions of 3 and 7 times a week showed the greatest effect on the cognitive improvement of the experimental groups in the post-intervention (change MoCA average: 3 and 5). However, it can be identified that the experimental group of Prokopenko et al. (2013) may have presented greater outcomes due to their sample’s higher level of education (10 years), combined with the intervention initiated two weeks after the stroke and daily individual training.

Both studies (Prokopenko et al., 2013; Faria et al., 2018) also showed improvements in the control groups. Comparatively, the control group in the study by Yeh et al. (2019) showed a low measure of change (0.25), even performing 36 sessions (2 or 3 per week), which may be related to the use of non-aerobic physical exercises and unstructured cognitive activities.

Although all experimental groups included in this review had post-intervention change (MoCA) scores, the study with the highest level of education and the shortest time to start rehabilitation after the stroke (Prokopenko et al., 2013) was the one that obtained the highest measure of post-intervention change (5 points of change in MoCA) in the experimental group. In general, it is possible to identify in this review that age, education, and post-stroke time were protective factors in cognitive training and able to predict the severity of post-stroke cognitive deficits (Mohd Zulkifly et al., 2016; Umarova et al., 2019; Mugisha et al., 2022). Therefore, a detailed presentation of the intervention process is valuable and follows.

### Characteristics of the interventions of the selected studies

Table 2 presents information concerning the interventions carried out in each study. This review included 4 studies that used computerized tools for motor and cognitive rehabilitation of stroke patients in the experimental groups. In addition, all computerized training was combined with other interventions such as standard therapy (Prokopenko et al., 2013; Kotov et al., 2020), occupational therapy (Faria et al., 2018), and aerobic exercise (Yeh et al., 2019).

The studies used different types of computerized intervention as follows: (i) the Reh@Task, a multifunctional virtual reality tool for reaching upper limbs and cognitive training in different settings and rehabilitation paradigms, focused on neutral stimulus and traditionally used in standard rehabilitation—symbols, numbers and letters (Faria et al., 2018); (ii) a complex multimodal stimulation program that included brain-computer neural interface technology with an arm exoskeleton used to reproduce de motion displayed in the images for the recovery of higher mental functions. Only this study used the computational tool in both groups (Kotov et al., 2020); (iii) individual training with computer programs: an original method of restoring cognitive functions developed by the study group itself focusing on four aspects of attention (sustained, selective, safe and shifting) and based on a computerized version of Schulte’s Tables with biological feedback and the possibility of help (Prokopenko et al., 2013); and (iv) the BrainHQ platform (Posit Science, 2022): an online brain gym scientifically designed to train and improve the brain and its cognitive functions (Yeh et al., 2019).

## Discussion

Our systematic review sought to investigate how and in which contexts CR proxies mediate the computerized training efficacy of patients 50 years or older affected by stroke and verify whether clinical variables such as stroke severity and age of occurrence could influence these results.

From the CR proxies and rehabilitation effectiveness, we identified that schooling associated with more intense conventional and computerized interventions (3–7 times a week) presented better results. Low schooling and aging were also identified as risk factors for post-stroke cognitive impairment in the study by Mohd Zulkifly et al. (2016).

In line with thesis results, investigations indicate that CR models the practicality of the neural network (processes and makes it flexible) and is related to the possibility of delaying the effect of the brain lesion on the affected person’s performance (Barulli and Stern, 2013; Umarova et al., 2021). Thus, CR proxies are used with a sense of operationalization related to the reserve. However, it is not a direct measure of CR (Umarova et al., 2021).

The study by Yeh et al. (2019) allowed us to identify that not only more years of schooling but its association with aerobic exercise and computerized cognitive training was presented as a determinant of cognitive improvement in stroke survivors.

Since physical activity is also considered a proxy for CR, achieved through the development of lifestyles (Barulli and Stern, 2013), strong evidence suggests that aerobic exercise is beneficial to improve aerobic fitness, walking speed, and endurance in people who have suffered a mild to moderate stroke (Pang et al., 2013). Furthermore, among the benefits related to physical conditioning, such as functionality, mood, and cardiovascular health, it was identified that aerobic exercise might potentiate the neuroplasticity process with more robust responses in training programs with moderate to high-intensity exercises (Penna et al., 2021). Finally, cognitive improvement is indicated in aerobic exercises combined with cognitive training, especially computerized training (Amorós-Aguilar et al., 2021).

In addition to schooling and aerobic activity, our review also suggests that ischemic stroke linked to post-stroke time, shorter-term and high-intensity training can mediate positive cognitive changes. Momosaki et al. (2016) identified that patients with ischemic stroke who underwent very early rehabilitation after thrombolysis obtained more favorable outcomes in functional independence.

In this sense, our systematic review advances the body of knowledge by observing that the time and intensity of cognitive training play a role in the cognitive benefit of the computerized training of patients affected by an ischemic stroke. Although the finding seems promising, a caveat is needed as current data do not allow generalizations. The numbers of studies, patients and comparable instruments were limited, making meta-analysis unfeasible. More research is needed to confirm whether short-term, high-intensity rehabilitation interventions are critical to more effective outcomes.

Studies indicate that ultra-early rehabilitation, that is, within 72 h after ischemic stroke, can facilitate brain neuroplasticity and functional reorganization (Stokowska et al., 2017; Pekny et al., 2019) with the emergence of new dendrites and axons such as those already shown in animal models (Liu et al., 2019). In line, the study by Liu et al. (2021) on the effects of different moments of early rehabilitation intervention in patients with acute ischemic stroke demonstrated that stroke patients in the ultra-early rehabilitation group showed greater improvement identified by the National Institutes of Health Stroke Scale, Body Mass Index, and simple Fugl-Meyer Assessment score at 1 month and 3 months, in comparison with the early rehabilitation program, which started between 72 h to 7 days after the stroke.

However, clinical factors such as lesion severity and size are also associated with cognitive impairment in stroke (Umarova et al., 2021). The possible moderation of these factors was not identified in our review. Despite this, there are relationships between motor, cognitive, and emotional impairments depending on the location and extent of the stroke lesion (Chen et al., 2000, 2022).

Regarding computerized cognitive training, it was identified in our systematic review that the impact of interventions led to cognitive improvements but equivalent to conventional rehabilitation identified by the MoCA screening measure and corroborated by other studies (Ozen et al., 2021; Mingming et al., 2022; Wiley et al., 2022). There was an investigation of the effect of virtual rehabilitation therapy on post-stroke cognition, and equivalence to other intervention modalities was identified, although it has the potential to improve motor, cognitive and physical deficits; the findings suggest that it is also necessary to investigate other variables that influence rehabilitation outcomes (Wiley et al., 2022).

The study by Ozen et al. (2021) presented computer game systems as recent and attractive interventions. There was an improvement in cognitive function in the group that used physical therapy plus occupational therapy (control). But both groups improved their quality of life by using experimental exercises, physical therapy, and specific tasks through computer games. In the motor aspect, there was an improvement in the hemiplegic upper limb in both groups and an improvement in the hand in the experimental group.

Although reliable and effective, conventional cognitive training is considered less efficient in providing instant and precise progressive control (Faria et al., 2018; Zhou et al., 2022). Perhaps its great strength is to be person-centered and aligned with the patients’ unique characteristics and circumstances to carry out the cognitive training. On the other hand, computer-assisted cognitive training with a high frequency of intervention has an advantage for physical and cognitive impairment, improving activities of daily living and functional status (Nie et al., 2022), being mainly important for cases of severe physical sequelae, which can limit the patients’ participation in rehabilitation, requiring an adaptable strategy to compensate for mobility difficulties (Prout et al., 2015). Moreover, in addition to providing immediate feedback, gamification makes it interactive and motivating, contributing to patients not giving up intervention (Faria et al., 2018; Mingming et al., 2022; Nie et al., 2022). That will be even more relevant for future generations born in the digital era.

### Limitations

Studies failed to report complete basic demographic variables such as age, gender, occupational status, schooling, clinical factors, and habits of those affected by stroke. Identifying which patients’ characteristics are correlated with the best outcomes allows for the development of more efficient cognitive computerized training. Noteworthily, some authors, when consulted, did not have such details to share, which are essential proxies of cognitive reserve, impeding the completion of meta-analyses. It is important to remind the readers that only four studies were included out of 19 which met the criteria. These articles were published in recent years (2013–2021), making the inaccessibility unjustified. It means that open science principles are not rigorously followed in this field of research.

Consequently, this systematic review was highly impacted by the limited information from the studies. Therefore, it is necessary to point out the importance of future research systematically reporting demographic and clinical information as well as adhering to the principles of open science, for instance, making demographic and clinical data about their patients properly stored and easily accessible (Wilkinson et al., 2016). It reduces questionable practices and makes them more transparent (Isaacowitz and Lind, 2019). Open science is particularly important for research involving the themes of aging and for developing systematic reviews and meta-analytic studies.

Another limitation is the lack of studies reporting comparative data between stroke patients and healthy controls. This is crucial to understand the impact of cultural and environmental aspects on performance, especially because not all studies use standardized measures.

A systematic investigation is fundamental to assess the effectiveness of computerized interventions compared to conventional ones, especially in patients who suffered a stroke (investigating the type of stroke, lesion size, duration, and intervention intensity). In addition, it seems essential to provide information about the rehabilitation center, its standards, infrastructure, and resources.

Although findings related to computational interventions are encouraging, caution must be taken since the number of studies included in the systematic review is small and the interventions varied, impeding a direct comparison between studies. Moreover, the level of digital literacy or familiarity with technological resources in the premorbid context of the patients is unclear, which might minimize the benefit of the intervention regardless of the quality of the latter. Further investigation with larger samples and longitudinal follow-ups are needed because it is uncertain if the intervention effect remains or could eventually appear later in time. More research with an emphasis on intervention time is also needed.

Interventions are often combined with pharmacological treatments to potentiate post-stroke recovery (Burgess and Alderman, 2013; Szelenberger et al., 2020; Mingming et al., 2022). A frequent problem is that not all studies report doses and duration of pharmacological treatments. Besides, they can be combined with premorbid treatments and non-prescribed substances. Although crucial, controlling this information is challenging and a common gap in the interpretation of findings.

## Conclusion

The present systematic review investigated the effects of rehabilitation with cognitive training, considering cognitive reserve proxies, and verifying the clinical variables that impact the results. All selected studies applied computerized cognitive training in the experimental group. Despite the limitation in the number of studies, there was an equivalence between computerized cognitive training and conventional therapies. Besides, findings confirm the mediation of higher education and intervention duration and emphasize the beneficial results of computerized interventions. Early intervention was extremely crucial to improve ischemic stroke outcomes. In addition, ischemic stroke and the time post-stroke appear to mediate the best outcome of the intervention, although clinical variables remain confounding factors.

## Data availability statement

The original contributions presented in this study are included in the article/supplementary material, further inquiries can be directed to the corresponding author.

## Author contributions

PF-F, SB-V, FR, and FS: research question and search strategies, data synthesis, and writing. PF-F and SB-V: search in literature, selection of studies and data extraction, and assessment of methodological quality. FR: resolve disagreement. FR and FS: proofreading and adding significant parts. All authors contributed to the article and approved the submitted version.
